# Cost of surviving sepsis: a novel model of recovery from sepsis in *Drosophila melanogaster*

**DOI:** 10.1186/s40635-016-0075-4

**Published:** 2016-01-21

**Authors:** Ata Murat Kaynar, Veli Bakalov, Silvia Martinez Laverde, Amélie I. F. Cambriel, Byoung-Hoon Lee, Atif Towheed, Alyssa D. Gregory, Steven A. R. Webb, Michael J. Palladino, Fernando A. Bozza, Steven D. Shapiro, Derek C. Angus

**Affiliations:** Clinical Research, Investigation, and Systems Modeling of Acute Illness (CRISMA) Laboratory, Department of Critical Care Medicine, University of Pittsburgh School of Medicine, 612 Scaife Hall, 3550 Terrace Street, Pittsburgh, 15261 PA USA; Department of Medicine, University of Pittsburgh School of Medicine, Pittsburgh, PA 15261 USA; Université Paris Descartes, 75270 Paris, Cedex 06 France; Department of Pulmonology and Allergy, Eulji University, Seoul, 139-711 Korea; Department of Pharmacology and Chemical Biology, Pittsburgh, PA 15260 USA; School of Population Health, University of Western Australia, Crawly, Perth, WA 6009 Australia; Instituto de Pesquisa Clínica Evandro Chagas, Fundação Oswaldo Cruz, Rio de Janeiro, 21040-360 Brazil; Present address: Center of Mitochondrial and Epigenomic Medicine, The Children’s Hospital of Philadelphia, Philadelphia, PA 19104 USA

**Keywords:** *Drosophila*, Sepsis, Recovery, Drosomycin, Insulin, Akt

## Abstract

**Background:**

Multiple organ failure, wasting, increased morbidity, and mortality following acute illness complicates the health span of patients surviving sepsis. Persistent inflammation has been implicated, and it is proposed that insulin signaling contributes to persistent inflammatory signaling during the recovery phase after sepsis. However, mechanisms are unknown and suitable pre-clinical models are lacking. We therefore developed a novel *Drosophila melanogaster* model of sepsis to recapitulate the clinical course of sepsis, explored inflammation over time, and its relation to impaired mobility, metabolic disturbance, and changes in lifespan.

**Methods:**

We used wild-type (WT), Drosomycin-green fluorescent protein (GFP), and NF-κB-luc reporter male *Drosophila melanogaster* 4–5 days of age (unmanipulated). We infected *Drosophila* with *Staphylococcus aureus* (infected without treatment) or pricked with aseptic needles (sham). Subsets of insects were treated with oral linezolid after the infection (infected with antibiotics). We assessed rapid iterative negative geotaxis (RING) in all the groups as a surrogate for neuromuscular functional outcome up to 96 h following infection. We harvested the flies over the 7-day course to evaluate bacterial burden, inflammatory and metabolic pathway gene expression patterns, NF-κB translation, and metabolic reserve. We also followed the lifespan of the flies.

**Results:**

Our results showed that when treated with antibiotics, flies had improved survival compared to infected without treatment flies in the early phase of sepsis up to 1 week (81 %, *p* = 0.001). However, the lifespan of infected with antibiotics flies was significantly shorter than that of sham controls (*p* = 0.001). Among infected with antibiotic sepsis survivors, we observed persistent elevation of NF-κB in the absence of any obvious infection as shown by culturing flies surviving sepsis. In the same group, geotaxis had an early (18 h) and sustained decline compared to its baseline. Geotaxis in infected with antibiotics sepsis survivors was significantly lower than that in sham and age-matched unmanipulated flies at 18 and 48 h. Expression of antimicrobial peptides (AMP) remained significantly elevated over the course of 7 days after sepsis, especially *drosomycin* (5.7-fold, *p* = 0.0145) on day 7 compared to that of sham flies. Infected with antibiotics flies had a trend towards decreased Akt activation, yet their glucose stores were significantly lower than those of sham flies (*p* = 0.001). Sepsis survivors had increased lactate levels and LDH activity by 1 week, whereas ATP and pyruvate content was similar to that of the sham group.

**Conclusions:**

In summary, our model mimics human survivors of sepsis with persistent inflammation, impaired motility, dysregulated glucose metabolism, and shortened lifespan.

**Electronic supplementary material:**

The online version of this article (doi:10.1186/s40635-016-0075-4) contains supplementary material, which is available to authorized users.

## Background

Sepsis is a significant global health problem, with more than 20 million patients affected annually and a direct economic costs calculated in $17 billion [1]. This disease imposes additional costs to the society, families, and patients, including lost days of work, absence from school, and long-term disabilities [2]. In recent years, a significant reduction in short-term mortality rates of patients with sepsis syndrome resulted in an increasing population of survivors [3]. Sepsis survivors frequently incur a dramatic decline in functional capacity and quality of life that can persist for years and carry a higher long-term risk of subsequent morbidity and mortality compared to age-matched controls, such as increased rates of cardiovascular events, new infections, cancer, and neurocognitive or muscular dysfunction, metabolic disturbances, and shortened lifespan [4–8]. Literature suggests that sustained inflammation is the driving force behind these metabolic and functional changes during the recovery from sepsis [9].

While the mechanisms of acute inflammatory response during the early phases of sepsis have been explored, mechanisms of resolution and tissue repair late in sepsis have not been evaluated. In the sepsis field, experimental models that mimic the long-term consequences of sepsis allowing us to explore the crosstalk between inflammation, metabolism, and functional recovery is lacking. Similarly, in *Drosophila*, infection models have been limited to host-pathogen interactions in the acute phase and do not address inflammatory, motility, metabolic disturbances, and changes in lifespan observed in patients surviving sepsis [10].

We therefore developed a *Drosophila* model of sepsis to explore inflammation, functional impairment, metabolic derangements, and lifespan in the recovery phase of sepsis by studying gene expression and protein translation of the inflammatory and metabolic pathways, motility, biochemical analyses of carbohydrate metabolism, and finally survival.

## Methods

### Experimental design

We developed a model of percutaneous infection in *D. melanogaster* to mimic humans recovering from sepsis and followed immune and functional outcomes over a course of 7 days and lifespan up to 60 days. We had four experimental groups, which were unmanipulated, sham, infected without treatment, and infected with antibiotics groups.

### *Drosophila melanogaster* strains and maintenance

The flies were raised at 23 °C, 60 % humidity, and 12-h light/dark cycle on standard cornmeal-yeast medium and changed every 3–5 days. We selected male flies 2–3 days after eclosion for experiments. We obtained *cec κB*-*luc* flies from Dr. Williams at the University of Pennsylvania [11]. Wild-type (WT) Canton S and Drosomycin-green fluorescent protein (GFP) reporter (Dipt-lacZ, Drs-GFP, y[1]/CyO) along with appropriate control flies were obtained from Bloomington stock.

### Fly infection

We prepared *Staphylococcus aureus* suspension in Luria-Bertani (LB) broth from frozen glycerol stock. Following an overnight culture, we transferred bacteria to a fresh tube to achieve the exponential growth phase (4–5 h) and then washed in phosphate-buffered serum (PBS). The bacterial pellet was resuspended in PBS to an optical density (OD) of 1.0 at 600 nm. At OD of 1.0, there were 1.67 × 10^6^ CFU of *S. aureus*. We anesthetized the flies with CO_2_ and pricked with a tungsten needle (0.01 mm at the tip and 0.25 mm across the needle body) into their thorax. The sham group was pricked with a sterile needle while the infected group had the needle dipped into the bacterial solution [10]. We pricked the flies at room temperature (23 °C) and placed the flies back into the vials to recover. We pricked the sham flies first to coat the tungsten needle with hemolymph to achieve consistent bacterial coating with the infection group.

### Treatment

We prepared linezolid-containing (500 μg/ml) fly food (cornmeal-yeast medium) by adding 50 mg of linezolid into 100 ml of liquefied food. We transferred unmanipulated, sham, and infected with antibiotics flies to antibiotic-containing vials immediately after infection and kept in these vials for 18 h before transferring the flies back to antibiotic-free vials. The infected without treatment group was placed into antibiotic-free new vials immediately after infection.

### Fly survival and lifespan

After infection, we assessed fly survival by visual inspection of living flies every 1 h (except night time) for the first 72 h. Infected without treatment flies started dying usually after 16 h of infection. We excluded flies that died within 6 h after inoculation from survival analysis. During lifespan observations over 60 days, we changed fly media and assessed survival every 2 days.

### Bacterial burden

We determined the bacterial burden of flies by sampling three groups of 10 files immediately, 6, 18, 36, 48, and 72 h and 7 and 20 days after inoculation and homogenized in LB broth. Serial 10× dilutions of final fly extracts were cultured on LB agar and colony forming units (CFU) were counted after 24-h incubation in 37 °C.

### NF-κB luciferase activity following infection

We measured luciferase activity in flies expressing *cec NF-κB reporter*. We prepared 96-well microwell plates for flies as described [11]. Each well contained two layers of food medium with the top layer containing luciferin. We added 300 μl of 5 % sucrose and 2 % agar solution to each well and allowed to solidify. Then, we added a 50-μl top layer to each well containing 5 % sucrose, 1 % agar, and 2 mM luciferin to detect NF-κB activation in infected, treated, and control flies. Similar to our other experiments, we added linezolid (500 μg/ml) to the top layer with the same concentration of sucrose, agar, and luciferin. We allowed plates to dry thoroughly to prevent flies from adhering to the condensates. Because luciferin is light sensitive, we avoided exposing plates to light more than necessary. Then, we applied a clear adhesive film to a 96-well microwell plate and made two holes per well for air exchange with a 25-gauge needle. We initially placed flies into vials containing 5 % sucrose and 2 % agar 2 days before experiment to acclimatize them to the food. Before needle pricking, we loaded flies into microwell plate under constant ambient light for 3 h to adjust to the new environment and to observe that they consume the luciferin substrate. After flies acclimatized and consumed luciferin, we anesthetized them with CO_2_ in groups of 24 and performed infection assay as described above. We returned each fly to its original well and resealed the microplate. We measured luminescence emission with a plate reader (SpectraMax M5, Molecular Devices, Sunnyvale, CA) at 25 °C every 10 min and 1.5 s per well. We followed flies for up to 72 h in the microwell plates. We expressed the luminescence data as relative luciferase activity per group of 24 surviving flies.

### Patterns of host response gene expression

We determined inflammatory gene expression, pattern recognition receptors, anti-microbial peptides, NF-kB, and insulin pathway members by collecting flies 6, 18, and 48 h and 1 week after needle pricking. We extracted RNA using RNeasy Mini kit (RNeasy Mini Kit/74104, QIAGEN, Germantown, MD). Gene expression of *Toll*, *PGRP-SD*, *defensin*, *drosomycin*, *metchnikowin*, *cecropin A*, *JNK*, *dorsal*, *Relish*, *Dif*, *IRS*, *FOXO*, *dAkt1*, *dPTEN*, *InR*, *TORC1*, *Glut-1*, and *Glut-3* were determined with quantitative real-time polymerase chain reaction (qRT-PCR) protocol using *actin-5C* as the housekeeping gene; all data were normalized to unmanipulated group. We used 20 flies per group in triplicates. Primers that we used in qRT-PCR were from the TaqMan® Gene Expression Assay: *toll*, Dm02151201_g1; *PGRP-SD*, Dm01840723_s1; *defensin*, Dm01818074_s1; *drosomycin*, Dm01822006_s1; *metchnikowin*, Dm01821460_s1*; cecropin A*, Dm02151846_gH; *JNK*(bsk), Dm01803999_g1; *dorsal*, Dm01810803_g1; *Relish*, Dm02134843_g1; *Dif* (Dorsal related immunity factor), Dm01810797_g1; *IRS* (Chico), Dm01803991_g1; *FOXO*, Dm02140207_g1; *dAkt1*, Dm02149559_g1; *dPTEN*, Dm01844965_g1; *TORC1*(CTRC), Dm01806284_s1; *InR*, Dm02136224_g1; *Glut-1*, Dm01821914_g1; *Glut3,* Dm02152390_s1; and *actin-5C*, Dm02361909_s1.

### Western immunoblotting for Drosomycin-GFP and Akt

We decapitated 10–12 flies and homogenized in 125 μl of RIPA buffer with phosphatase and proteinase inhibitors. Samples were then heated at 95 °C for 5 min, sonicated, and loaded into the wells of an 18 % SDS-PAGE gel. We used GFP antibody (Santa Cruz Biotechnology Inc., TX) at 1:2500, Phospho-Akt (Ser473) Antibody #9271, and Akt Antibody #9272 (Cell Signaling Technology, Danvers, MA) at 1:5000 dilutions. Anti-ATP- α (a5-c antibody, Developmental Studies Hybridoma Bank, University of Iowa, USA) was used as a loading control. ATP-α is a nuclear encoded plasma membrane protein (the catalytic subunit of the Na^+^/K^+^ ATPase). Secondary detection was performed using anti-mouse (1:4000) or anti-rabbit (1:5000) (Bio-Rad, Hercules, CA) HRP conjugated antibodies.

### Rapid iterative negative geotaxis (RING)

We joined two empty polystyrene vials by tape vertically facing each other forming an 18.5-cm-long tube [12]. We transferred groups of 20 flies into the vials and allowed to acclimatize to the new setting for 5 min before conducting the assay. Flies were gently tapped down to the bottom of the vial for 10 s with the same interval and strength by the same operator throughout the whole experiment. Pictures of the flies were taken with a digital camera at 5 s. We repeated each geotaxis experiment six times, allowing for 1-min rest periods between each trial and pictures were analyzed by counting the number of flies that climb above the 10-cm mark in 5 s after the tap. We calculated the average of the number of flies crossing the 10-cm threshold and expressed the results as percentage of the total number of flies in the tube (=% climbing index). Each geotaxis experiment was performed 1 h before the needle pricking (0 h baseline) and at 18, 48, 72, and 96 h after needle pricking. The data are presented as percent of the baseline at time 0 h. All of the control groups were kept in antibiotic-containing media.

### Protein and metabolite measurements

We measured total protein (Bio-Rad DC), glucose (GAGO20-1KT, Sigma-Aldrich), glycogen (A1602, Sigma-Aldrich), lactate (MAK064-1KT, Sigma-Aldrich), pyruvate (MAK071-1KT, Sigma-Aldrich), and ATP (MAK190-1KT, Sigma-Aldrich) concentrations and lactate dehydrogenase activity (MAK066-1KT, Sigma-Aldrich) in flies at 18, 48, and 168 h (1 week) after infection in triplicate vials with 10–15 flies in each vial, according to the manufacturer’s recommendations. We decapitated flies to minimize red eye color effect on outcomes of the colorimetric assays.

### Statistical analysis

Kaplan-Meyer survival analysis was performed using GraphPad Pad 6 (La Jolla, CA). Statistical analysis between different groups was accomplished with *t* test analysis and timed changes in geotaxis and NF-κB with ANOVA using GraphPad Pad 6.

## Results

### Sepsis in *Drosophila*

#### Antibiotic treatment improves short-term survival and decreases bacterial burden in infected flies

**Figure 1:**
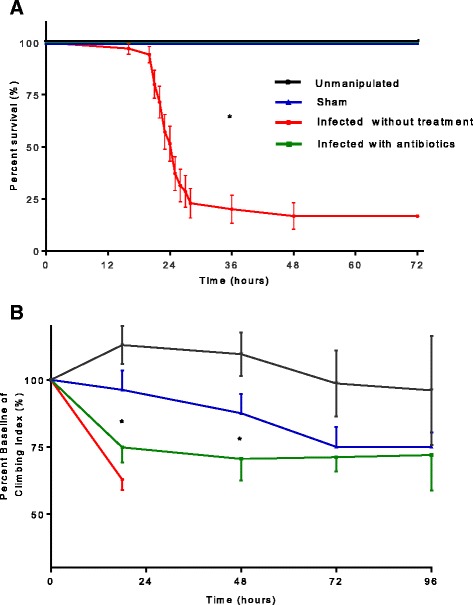
**a** Survival in *Drosophila melanogaster* after septic injury with *Staphylococcus aureus.* Flies were infected using needle pricking assay with *S. aureus*; survival was assessed by observing flies every hour for the first 72 h from 8 a.m. until 11 p.m. Of the infected without treatment flies, 75 % died by the 28th hour after infection; infected with antibiotics group received oral linezolid for 18 h and showed improved survival without any death due to infection in the first 72 h after infection. Sham group pricked with sterile needle showed no death due to pricking-related trauma. We used 25 flies in each group; survival experiment was repeated three times for the Kaplan-Meyer survival analysis (*p < 0.05). **b** Geotaxis in *Drosophila melanogaster* after septic injury with *Staphylococcus aureus.* Each geotaxis experiment was performed 1 h before the needle pricking (0-h baseline) and at 18, 48, 72, and 96 h after needle pricking. Geotaxis in sham flies was significantly lower at 72 h compared to its baseline. Infected with antibiotics group showed significantly lower geotaxis compared to baseline at 18, 48, and 72 h. While there was significant difference in geotaxis between sham and infected with antibiotics groups at 18 and 48 h, this difference was lost by 72 h. Geotaxis of unmanipulated healthy flies remained at the baseline through experiment. We repeated each geotaxis experiment six times (*p < 0.05)

After the induction of infection with *S. aureus* at OD of 1, we observed 83 % mortality by 72 h in the infected without treatment group. We successfully reversed mortality in infected flies by feeding the animals with oral linezolid in the infected with antibiotics group (*p* < 0.001) (Fig. 1a). Among those receiving no antibiotics, survival rate was too low to permit stable statistical analysis after 72 h. Age-matched unmanipulated control and sham flies had no mortality by 72 h after surviving sepsis (Fig. 1a). The antibiotic-containing food had no impact on the survival of flies as compared to the regular food.

Flies in the infected with antibiotics group not only had higher survival but also had lower bacterial burden 24 h after infection (*p* < 0.0001) (Additional file 1: Figure S1). The weight of flies was not different between groups over a week period (Additional file 2: Figure S2).

### Impaired motility following sepsis

#### Rapid iterative negative geotaxis (RING) is decreased in flies surviving sepsis

The geotactic ability of unmanipulated flies remained unchanged throughout the 4 days of experimental period. However, geotaxis in sham flies was significantly lower at 72 h compared to its baseline. Infected with antibiotics group showed significantly lower geotaxis compared to baseline at 18, 48, and 72 h. While there was significant difference in geotaxis between sham and infected with antibiotics groups at 18 (sham, 96.2 % vs. infected with antibiotics, 74.8, *p* = 0.028) and 48 (sham, 87.5 % vs. infected with antibiotics group, 70.5 %, *p* = 0.039) hours, this difference was lost by 72 h. Geotaxis of unmanipulated healthy flies remained at the baseline through experiment (Fig. 1b).

### Inflammation following sepsis

#### Persistent NF-κB activation is present in flies surviving sepsis

**Fig. 2:**
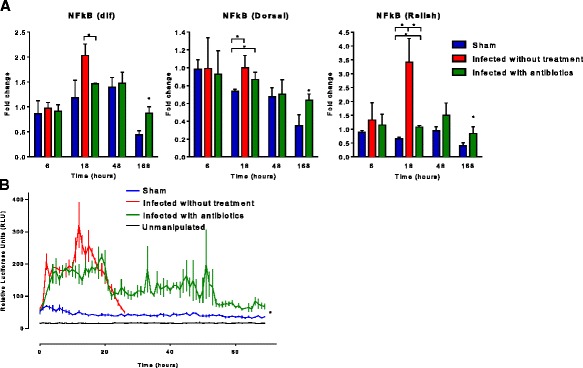
NF-κB activity in *Drosophila melanogaster* after septic injury with *Staphylococcus aureus*. **a** We determined NF-κB expression at 6, 18, 48, and 168 h (1 week) after needle pricking with qRT-PCR. Expression of *dif* and *Dorsal* 18 h after infection was upregulated in infected with antibiotics group and was significantly higher compared to that of sham; expression of *Relish* was upregulated in all three groups with significant difference; all three NF-κB components (*dorsal*, *Dif*, *Relish*) remained elevated by 1 week following survival from sepsis (*p < 0.05). **b** Luciferase activity in live flies expressing *NF-κB luciferase reporter* was measured for 72 h after infection. Flies demonstrated elevated levels of NF-κB activation in both infected without treatment and infected with antibiotics groups in the first 24 h and sustained elevation of NF-κB activity up to 72 h in infected with antibiotics flies, while activity in sham group was not upregulated after pricking (*p < 0.05)

The centrally located signaling molecule for pro-inflammatory signaling, NF-κB, was upregulated in infected without treatment flies. At transcriptional level, all three NF-κB components (dorsal, Dif, Relish) remained elevated by 1 week following survival from sepsis in the infected with antibiotics group (Fig. 2a). Luciferase activity in flies expressing *cec* NF-κB reporter demonstrated elevated levels of NF-κB activation up to 72 h in the infected with antibiotics group, while activity in the sham group was not upregulated after pricking (*p* < 0.0001) (Fig. 2b). Both groups were on antibiotic-containing media.

### Inflammatory receptors and effectors following sepsis

#### Pattern recognition receptors (PRR) and antimicrobial peptides (AMP) are persistently elevated in flies surviving sepsis

The genes encoding for PRR were upregulated as early as 6 h after infection. Gram-positive bacterial recognition receptors (PGRP-SD) remained similarly elevated between the infected without treatment as well as infected with antibiotics groups. Expression of Toll was significantly higher in the infected with antibiotics and infected without treatment compared to that in sham.

**Fig. 3:**
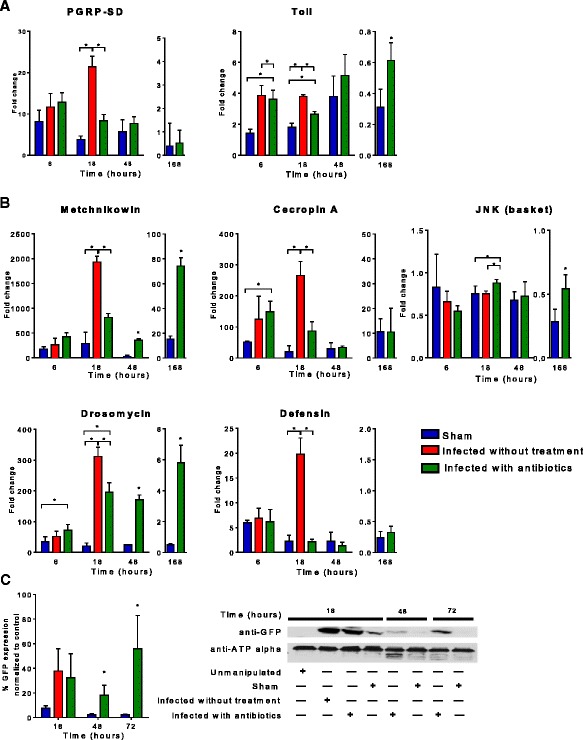
Inflammatory genes expression in *Drosophila melanogaster* after septic injury with *Staphylococcus aureus*. We determined inflammatory gene expression, pattern recognition receptors, antimicrobial peptides at 6, 18, 48, and 168 h (1 week) after needle pricking with qRT-PCR (*p < 0.05). **a** *PGRP-SD* and *Toll* were upregulated as early as 6 h after infection. By 18 h, *PGRP-SD* and *Toll* expression in infected without treatment flies were significantly elevated compared to infected with antibiotics flies. Toll remained elevated 48 h after infection with treatment; however, there was no difference between infected with antibiotics and sham; 1 week after infection expression of *Toll* was downregulated. **b** Antimicrobial peptides (AMP) were upregulated by 6 h in a similar way between infected without treatment vs. infected with antibiotics groups. By 18 h, the infected without treatment flies had significantly elevation of *drosomycin*, *metchnikowin*, *defensin*, JNK, and *cecropin A. Drosomycin* and *metchnikowin* remained significantly elevated in the survivors 48 h and 7 days after surviving sepsis. **c** Expression of *Drs*-*GFP* construct was detected at baseline and significantly increased as early as 18 h following infection using Western immunoblot. While its expression came back to baseline in sham flies by 48 h, the infected with antibiotics group had sustained elevation of *Drs-GFP* expression by 72 h

By 18 h, PGRP-SD and Toll expression in infected without treatment flies were significantly elevated compared with infected with antibiotics flies (PGRP-SD: infected without treatment, 21.4-fold; infected with antibiotics, 8.34-fold, *p* = 0.0033; Toll: infected without treatment, 2.6-fold; infected with antibiotics, 1.8-fold, *p* = 0.0016). However, there was a significant difference in Toll expression between infected with antibiotics and *sham* 1 week after infection (infected with antibiotics, 0.61-fold and sham 0.31, *p* = 0.032) (Fig. 3a).

The genes encoding for AMP were all upregulated by 6 h, the difference between sham and infected with antibiotics flies were significant for Drosomycin and Cecropin A expression. By 18 h, the infected without treatment flies had significantly elevated levels of *drosomycin*, *metchnikowin, defensin, and cecropin A* compared to infected with antibiotics as well as sham flies. *Drosomycin* and *metchnikowin* remained significantly elevated in infected with antibiotics survivors 48 h and 7 days after infection compared to sham flies despite downregulation of the PGRP-SD and Toll (Fig. 3b)*.*

The expression for JNK kinase (c-Jun N-terminal kinases) was unchanged between groups, yet by 1 week, the downregulation was more pronounced in the sham group compared to the infected with antibiotics group.

The expression of the Drosomycin-GFP construct (~25 kDa) was detected at baseline and significantly increased as early as 18 h following infection using Western immunoblot (Fig. 3c). While its expression came back to baseline in sham flies by 48 h, the infected with treatment group had sustained elevation of Drosomycin-GFP expression by 72 h.

### Metabolic pathways following sepsis

#### Insulin signaling pathway elements are persistently elevated in flies surviving sepsis

By 18 h, InR was upregulated and significantly higher in the infected without treatment group compared to sham (*p* < 0.05). Akt, a serine/threonine-specific protein kinase playing key roles in multiple cellular processes such as glucose metabolism, apoptosis, cell proliferation, transcription, and cell migration, was also upregulated by 18 h with significant difference between all three groups (*p* < 0.05).

**Fig. 4:**
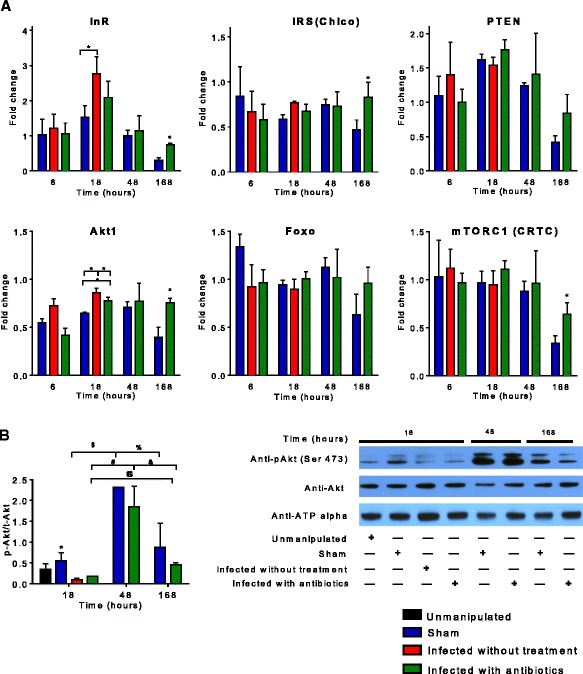
Insulin signaling pathway genes expression in *Drosophila melanogaster* after septic injury with *Staphylococcus aureus.* **a** We determined insulin pathway gene (InR, IRS, PTEN, Akt1, Foxo, and mTOR) expression at 6, 18, 48, and 168 h (1 week) after needle pricking. By 18 h, InR was upregulated and significantly higher in infected without treatment group compared to sham and Akt was upregulated with significant difference between all three groups. By 48 h in infected with antibiotics and sham groups, all insulin pathway genes showed sustained elevation of expression without significant difference between groups. In infected with antibiotics group 1 week after infection, levels of InR, IRS, Akt, and mTORC1 were upregulated and significantly higher than those in the sham group. (*p < 0.05). **b** We determined phosphorylated-Akt/total-Akt ratio with Western immunoblot at 6, 18, 48, and 168 h after needle pricking. Akt phosphorylation significantly decreased by 18 h in infected without treatment and infected with antibiotics compared to sham (*asterisk*). Akt phosphorylation significantly increased in sham between 18 and 48 h (*dollar sign*), yet then it was significantly lower at 168 h compared to 48 h (*percent*). Akt phosphorylation at 168 h after infection was not significantly different compared to 18 h after infection. Akt phosphorylation significantly increased in infected with antibiotics group 48 h after needle pricking compared to 18 h (*number sign*), yet then it was significantly lower at 168 h compared to 48 h (*ampersand*). Akt phosphorylation significantly increased in infected with antibiotics group 168 h after needle pricking compared to 18 h after infection (*commercial at sign*)

Levels of InR, Akt, IRS, and mTORC1—a nutrient/energy/redox sensor—were upregulated by 1 week after infection in infected with antibiotics group and significantly higher than those in the sham group (Fig. 4a).

Despite the increased gene expression pattern for IIS and Akt, the key signaling molecule in IIS, there was a trend towards decreased Akt phosphorylation in infected with antibiotics flies compared to sham even 1 week after surviving sepsis (Fig. 4b).

### Glucose metabolism is dysregulated in flies surviving sepsis

**Fig. 5:**
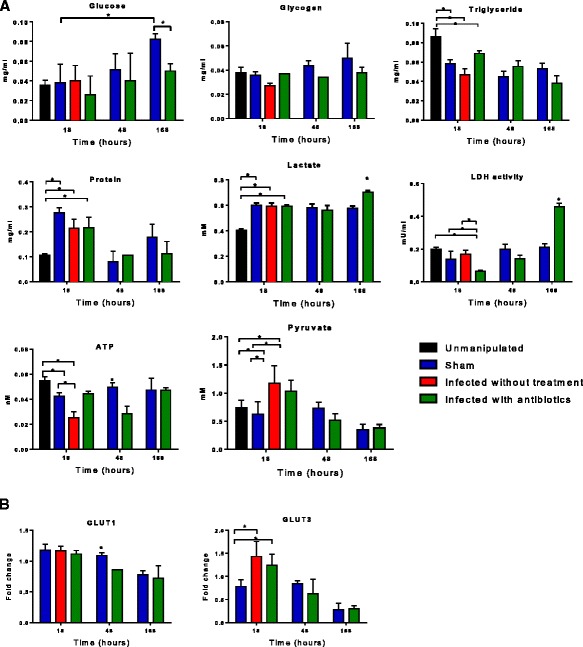
Carbohydrate metabolites in *Drosophila melanogaster* after septic injury with *Staphylococcus aureus.* **a** We measured glucose and glycogen concentrations at 18, 48, and 168 h in triplicate. We determined glycogen by measuring glucose level after digesting glycogen with amyloglucosidase into free glucose. Level of glucose significantly increased by 168 h in sham flies compared to 18 h. By 168 h, glucose content was significantly lower in infected with antibiotics flies compared to the sham group. Glycogen, triglyceride, protein, and pyruvate levels at 168 h were similar between the two groups. Lactate and LDH activity were significantly elevated by 168 h after surviving sepsis (infected with antibiotics group) (*p < 0.05). Interestingly, the ATP content was significantly lower in infected with antibiotics flies by 48 h compared to sham; however, this difference was lost by 168 h. **b** There was no difference in the expression of Glut-1 and Glut-3 by 168 h between sham and infected with antibiotics flies

Glucose content increased in the sham group after 1 week of sterile needle injury, whereas the infected with antibiotics flies surviving sepsis had significantly lower glucose stores at the same time point (*p* < 0.05) (Fig. 5a). The glycogen, protein, and triglyceride content were not different between the two groups by 1 week. Interestingly, lactate increased significantly in infected with antibiotics compared to sham group by 1 week and this was paralleled with a significantly increased activity of LDH in infected with antibiotics flies (*p* < 0.05). There was no difference in measured pyruvate and ATP content 1 week after surviving sepsis.

Gene expression of glucose transporters type 1 (GLUT-1) was unchanged by 18 h and was lower in the infected with antibiotics group compared with the sham group by 48 h. The expression of GLUT-3 was upregulated in infected without treatment and infected with antibiotics flies by 18 h compared to that in the sham group (Fig. 5b). However, the expression levels were similar by 1 week between groups for both transporters studied.

### Lifespan is significantly decreased in flies surviving sepsis

**Fig. 6:**
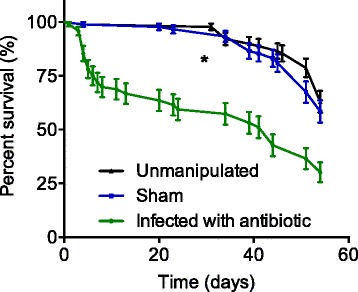
Lifespan of *Drosophila melanogaster* after septic injury with *Staphylococcus aureus. Drosophila* surviving sepsis induced by *Staphylococcus aureus* and treated with linezolid had significantly shorter lifespan compared to sham and unmanipulated flies over the course of 60 days (*p < 0.0001). The food intake has not been measured in different groups; however, the weight of flies by 1 week was unchanged in between groups (Additional file 2: Figure S2). Sham and unmanipulated flies had a similar lifespan. The antibiotic-containing food had no impact on the survival of flies as compared to the regular food

After infection with *S. aureus* and treatment with linezolid, the infected with antibiotics group of flies had no mortality within the first 72 h; however, they started to die after day 3 and by 1 week, the mortality was ~19 %. Despite surviving sepsis in the first week, sepsis survivors had a significantly decreased lifespan compared to sham and unmanipulated flies (*p* < 0.0001). Sham and unmanipulated flies had a similar lifespan (Fig. 6). The antibiotic-containing food had no impact on the survival of flies as compared to the regular food.

## Discussion

We developed a model of sepsis in *Drosophila*, in which despite elimination of bacterial pathogens and reversal of lethality with oral antibiotic administration, survivors exhibited several features consistent with that seen in humans. Notably, persistent inflammation with elevated NF-κB levels even 1 week after recovering from sepsis along with functional mobility defects including impaired geotaxis; sustained transcription of IIR signaling pathway components (InR, IRS, Akt, and mTORC1), impaired glucose metabolism, increased lactate production and LDH activity; and a shorter lifespan in flies surviving sepsis compared to sham [2, 5, 13].

The orally available antibiotic (linezolid) restored survival during the acute phase after sepsis. However, we observed functional derangements of geotaxis during recovery phase, similar to functional impairments seen in patients surviving sepsis [2]. The decline in geotaxis had a trend towards normalization after 72 h, suggesting a partially reversible phenotype [14]. Neuromuscular impairment following sepsis reduces quality of life and increases long-term mortality of sepsis survivors and has been associated with the degree of inflammatory and metabolic responses [2]. We similarly observed a shortened lifespan in infected with antibiotics sepsis survivors along with limited glucose stores compared to sham flies.

NF-κB, remained elevated in *Drosophila* surviving sepsis, with an early peak followed by a lower, yet sustained NF-κB over a 3-day period through continually monitoring *cec κB*-*luc* luciferase activity [11]. The transcription for NF-κB components (dif, Dorsal, Relish) had a similar pattern and this was sustained at 1 week after sepsis in survivors, whereas the sham controls had significantly lower expression of NF-κB components by 1 week. Such a pattern of peaked inflammation followed by low levels of sustained inflammation has recently been proposed in mammalian models of infection [15].

Following induction of sepsis, simultaneous with the NF-κB peak activity, we measured a peak of antimicrobial peptide expression. While the infection was cleared on day 2 and the difference in transcription of pattern recognition receptors (PGRP-SD and Toll) between sham and infected with antibiotics sepsis survivors was downregulated, antimicrobial peptide transcription was still significantly elevated and sustained for a week. Especially, translation of Drosomycin was significantly elevated by 72 h in infected with antibiotics sepsis survivors and its transcription was sustained by 1 week after recovery from sepsis [16, 17].

A robust AMP response is beneficial to the organism acutely; however, questions arise regarding why AMPs are sustained beyond the acute phase of sepsis in the absence of Toll-mediated signaling. Experimental data suggests that a secondary infection would be warded off easier if there were a non-lethal infection preceding that [18, 19]. However, sustained inflammation in patients recovering from sepsis increases the risk of organ failure [8]. Similarly, we showed in a model of atherosclerosis and surviving sepsis that persistent local and systemic inflammatory response accelerated atheroma burden in mice despite the fact that they appeared clinically normal [7]. The sustained inflammation thus may lead to shortened lifespan of survivors of sepsis. Our current model as well as previous data suggests that immune competence is an important determinant of fitness, but costly to the host requiring trade-offs with other energy demanding processes such as growth and reproduction [15, 18–22]. When we measured glucose content in flies surviving sepsis, flies surviving sepsis had significantly lower stores of glucose compared to sham and increased lactate levels, yet similar ATP contents in both groups [15, 23–27]. Sepsis, the epitome of a complex relation between pathogen and host, is energetically expensive and results in maladaptive allocation of resources away from growth to antimicrobial response, akin to the preferred aerobic glycolysis over oxidative phosphorylation seen in cancer cells [7, 23, 28, 29].

Similar to the metabolic switch from oxidative phosphorylation to aerobic glycolysis in cancer cells, during bacterial, mycobacterial, or parasitic infections, insulin signaling components (FOXO, PI3K, and Akt) contribute to metabolic wasting hastening the death of the host [21, 22, 30–32]. Additionally, Akt, JNK, mTOR, and HIF-1α regulate innate immune memory (“*trained immunity*”) by switching from the oxidative phosphorylation to aerobic glycolysis [15, 22, 33]. Such innate immune memory then sustains a pro-inflammatory state, a trade-off between health span and lifespan suggesting an overarching role of insulin signaling pathways in regulating inflammation [15]. In our model, glucose stores were depleted and lactate elevated in survivors of sepsis and the same group had significantly shorter lifespan mimicking patients surviving sepsis [34].

Model organism *D. melanogaster* is an important tool to understand acute response to infections but has not been used to identify damage and repair pathways during recovery from sepsis. Our model that mimics the recently described persistent inflammation, immunosuppression, and catabolic syndrome (PICS) observed in survivors of sepsis, however, has provided more unresolved questions if not some limitations [19, 35]. First, we did not measure the caloric intake in *Drosophila*; however, they had similar weights by 1 week. Second, linezolid inhibits mitochondrial protein synthesis, yet we used the same antibiotic containing food for sham and infected animals [36]. Lastly, we did not measure DAMPs, address the epigenetic changes, activity of pyruvate kinase M2, and sirtuins in the path of metabolic switch, nor interrogate the intricate relations between TOR, NF-κB, and Akt pathways [37–39].

## Conclusions

**Fig. 7:**
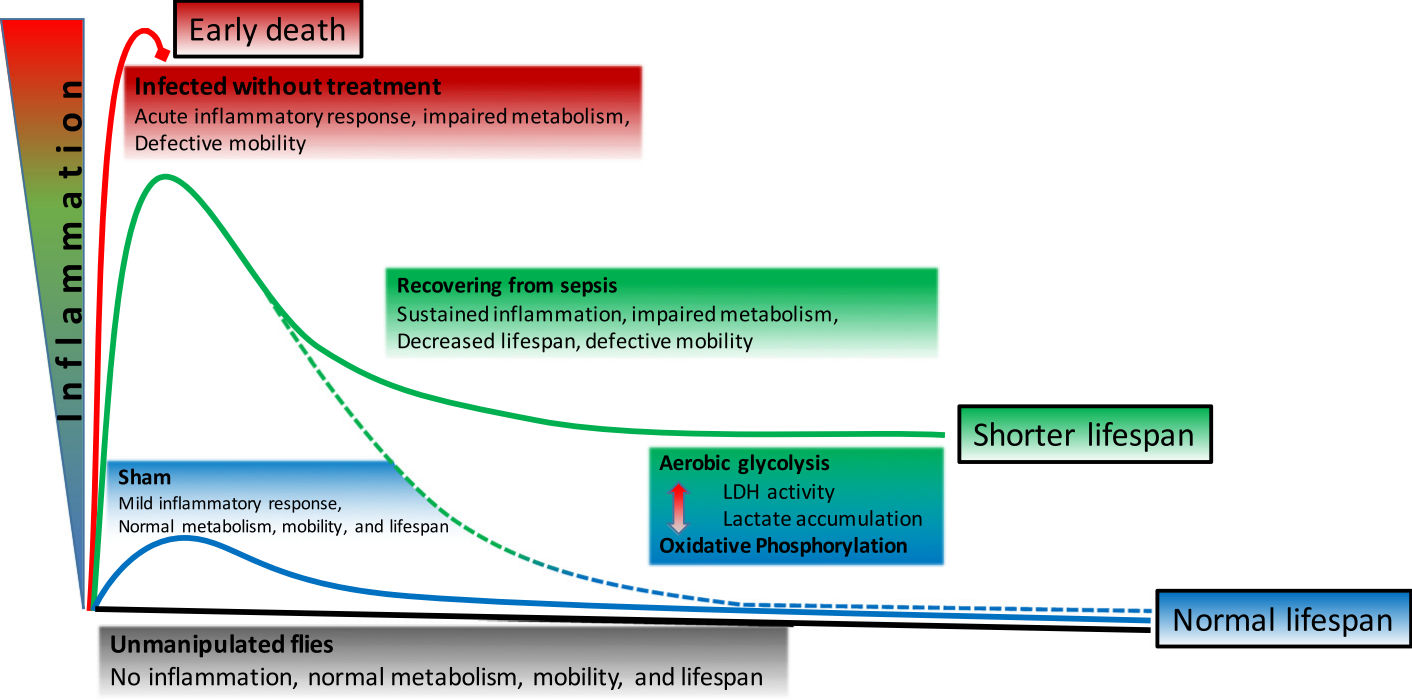
Summary illustration: interplay between impaired carbohydrate metabolism and sustained inflammation in *Drosophila melanogaster* surviving sepsis. *Drosophila* surviving sepsis induced by *Staphylococcus aureus* and treated with linezolid had impaired glucose metabolism with increased lactate production, increased LDH activity, sustained inflammation, and significantly shorter lifespan. The switch between oxidative phosphorylation and aerobic glycolysis could be the metabolic regulator of inflammation

In summary, we here developed a *Drosophila* model of recovery from sepsis mimicking human survivors of sepsis with persistent inflammation, impaired motility, dysregulated glucose metabolism, and shortened lifespan (Fig. 7). This model will open new avenues for researchers to test hypotheses about the crosstalk between inflammatory and metabolic pathways as well as impact of metabolic manipulations on inflammation and survival during the recovery phase of sepsis [33, 40].

## Additional files

Additional file 1: Figure S1. Bacterial load of *Drosophila melanogaster* after septic injury with *Staphylococcus aureus. Drosophila* were harvested each 24 h following the induction of sepsis induced by *S. aureus*. Flies surviving sepsis had 5.2 CFU/fly by 1 week with no significant difference compared to the sham group. (PDF 66 kb) Additional file 2: Figure S2. Weight of flies over a course of 1 week after sepsis. *Drosophila* were harvested at baseline, 18, 48, and 168 h after induction of sepsis and weighed in groups of five flies in triplicate. The weights (0.8 ± 0.053 μg/fly) were unchanged within and between groups over a 1-week period following sepsis. (PDF 67 kb)

## References

[1] Angus DC, Linde-Zwirble WT, Lidicker J (2001). Epidemiology of severe sepsis in the United States: analysis of incidence, outcome, and associated costs of care. Crit Care Med.

[2] Iwashyna TJ, Ely EW, Smith DM (2010). Long-term cognitive impairment and functional disability among survivors of severe sepsis. JAMA.

[3] Kaukonen KM, Bailey M, Suzuki S (2014). Mortality related to severe sepsis and septic shock among critically ill patients in Australia and New Zealand, 2000-2012. JAMA.

[4] Yende S, Angus DC (2007). Long-term outcomes from sepsis. Curr Infect Dis Rep.

[5] Yende S, D’Angelo G, Mayr F (2011). Elevated hemostasis markers after pneumonia increases one-year risk of all-cause and cardiovascular deaths. PLoS One.

[6] Remick DG, Bolgos G, Copeland S (2005). Role of interleukin-6 in mortality from and physiologic response to sepsis. Infect Immun.

[7] Kaynar AM, Yende S, Zhu L (2014). Effects of intra-abdominal sepsis on atherosclerosis in mice. Crit Care.

[8] Kellum JA, Kong L, Fink MP (2007). Understanding the inflammatory cytokine response in pneumonia and sepsis: results of the Genetic and Inflammatory Markers of Sepsis (GenIMS) Study. Arch Intern Med.

[9] Boonen E, Van den Berghe G (2014). Endocrine responses to critical illness: novel insights and therapeutic implications. J Clin Endocrinol Metab.

[10] Apidianakis Y, Rahme LG (2009). Drosophila melanogaster as a model host for studying Pseudomonas aeruginosa infection. Nat Protoc.

[11] Kuo TH, Handa A, Williams JA (2012). Quantitative measurement of the immune response and sleep in Drosophila. J Vis Exp..

[12] Gargano JW, Martin I, Bhandari P (2005). Rapid iterative negative geotaxis (RING): a new method for assessing age-related locomotor decline in Drosophila. Exp Gerontol.

[13] Quartin AA, Schein RM, Kett DH (1997). Magnitude and duration of the effect of sepsis on survival. Department of Veterans Affairs Systemic Sepsis Cooperative Studies Group. JAMA.

[14] Petersen AJ, Katzenberger RJ, Wassarman DA (2013). The innate immune response transcription factor relish is necessary for neurodegeneration in a Drosophila model of ataxia-telangiectasia. Genetics.

[15] Cheng SC, Quintin J, Cramer RA (2014). mTOR- and HIF-1alpha-mediated aerobic glycolysis as metabolic basis for trained immunity. Science.

[16] Lemaitre B, Reichhart JM, Hoffmann JA (1997). Drosophila host defense: differential induction of antimicrobial peptide genes after infection by various classes of microorganisms. Proc Natl Acad Sci U S A.

[17] Yang WY, Wen SY, Huang YD (2006). Functional divergence of six isoforms of antifungal peptide Drosomycin in Drosophila melanogaster. Gene.

[18] Delano MJ, Thayer T, Gabrilovich S (2011). Sepsis induces early alterations in innate immunity that impact mortality to secondary infection. J Immunol.

[19] Pham LN, Dionne MS, Shirasu-Hiza M (2007). A specific primed immune response in Drosophila is dependent on phagocytes. PLoS Pathog.

[20] Lochmiller R, Deerenberg C (1999). Trade-offs in evolutionary immunology: just what is the cost of immunity?. Oikos.

[21] Chambers MC, Song KH, Schneider DS (2012). Listeria monocytogenes infection causes metabolic shifts in Drosophila melanogaster. PLoS One.

[22] DiAngelo JR, Bland ML, Bambina S (2009). The immune response attenuates growth and nutrient storage in Drosophila by reducing insulin signaling. Proc Natl Acad Sci U S A.

[23] Demas GE, Chefer V, Talan MI (1997). Metabolic costs of mounting an antigen-stimulated immune response in adult and aged C57BL/6 J mice. Am J Physiol.

[24] Ayres JS, Schneider DS (2009). The role of anorexia in resistance and tolerance to infections in Drosophila. PLoS Biol.

[25] Raberg L, Vestberg M, Hasselquist D (2002). Basal metabolic rate and the evolution of the adaptive immune system. Proc Biol Sci.

[26] Hanssen SA, Hasselquist D, Folstad I (2004). Costs of immunity: immune responsiveness reduces survival in a vertebrate. Proc Biol Sci.

[27] Vanzant EL, Lopez CM, Ozrazgat-Baslanti T (2014). Persistent inflammation, immunosuppression, and catabolism syndrome after severe blunt trauma. J Trauma Acute Care Surg.

[28] Slack C, Giannakou ME, Foley A (2011). dFOXO-independent effects of reduced insulin-like signaling in Drosophila. Aging Cell.

[29] Vander Heiden MG, Cantley LC, Thompson CB (2009). Understanding the Warburg effect: the metabolic requirements of cell proliferation. Science.

[30] Lazzaro BP, Galac MR (2006). Disease pathology: wasting energy fighting infection. Curr Biol.

[31] Hwangbo DS, Gershman B, Tu MP (2004). Drosophila dFOXO controls lifespan and regulates insulin signalling in brain and fat body. Nature.

[32] Zhang W, Patil S, Chauhan B (2006). FoxO1 regulates multiple metabolic pathways in the liver: effects on gluconeogenic, glycolytic, and lipogenic gene expression. J Biol Chem.

[33] Doherty JR, Cleveland JL (2013). Targeting lactate metabolism for cancer therapeutics. J Clin Invest.

[34] Libert S, Chao Y, Zwiener J (2008). Realized immune response is enhanced in long-lived puc and chico mutants but is unaffected by dietary restriction. Mol Immunol.

[35] Gentile LF, Cuenca AG, Efron PA (2012). Persistent inflammation and immunosuppression: a common syndrome and new horizon for surgical intensive care. J Trauma Acute Care Surg.

[36] Garrabou G, Soriano A, Lopez S (2007). Reversible inhibition of mitochondrial protein synthesis during linezolid-related hyperlactatemia. Antimicrob Agents Chemother.

[37] Dan HC, Ebbs A, Pasparakis M (2014). Akt-dependent activation of mTORC1 complex involves phosphorylation of mTOR (mammalian target of rapamycin) by IkappaB kinase alpha (IKKalpha). J Biol Chem.

[38] Palsson-McDermott EM, Curtis AM, Goel G (2015). Pyruvate kinase M2 regulates Hif-1alpha activity and IL-1beta induction and is a critical determinant of the warburg effect in LPS-activated macrophages. Cell Metab.

[39] Liu TF, Vachharajani VT, Yoza BK (2012). NAD + -dependent sirtuin 1 and 6 proteins coordinate a switch from glucose to fatty acid oxidation during the acute inflammatory response. J Biol Chem.

[40] Dietl K, Renner K, Dettmer K (2010). Lactic acid and acidification inhibit TNF secretion and glycolysis of human monocytes. J Immunol.

