# Supplementary figures and images for: Cost of surviving sepsis: a novel model of recovery from sepsis in Drosophila melanogaster
Source: Intensive Care Med Exp. 2016 Jan 21;4:4. doi: 10.1186/s40635-016-0075-4 (PMC4720623; doi:10.1186/s40635-016-0075-4)

**S1**

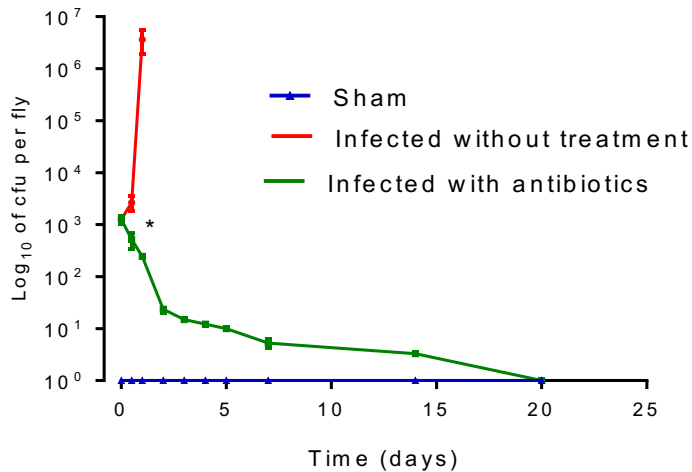

Supplement: Additional file 1: Figure S1. — Bacterial load of Drosophila melanogaster after septic injury with Staphylococcus aureus. Drosophila were harvested each 24 h following the induction of sepsis induced by S. aureus. Flies surviving sepsis had 5.2 CFU/fly by 1 week with no significant difference compared to the sham group. (PDF 66 kb) [file 40635_2016_75_MOESM1_ESM.pdf]

**S2**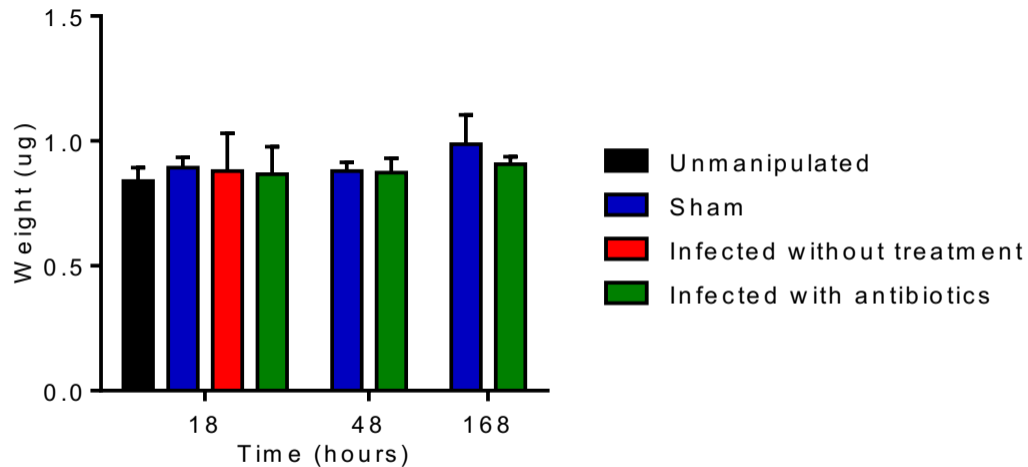

Supplement: Additional file 2: Figure S2. — Weight of flies over a course of 1 week after sepsis. Drosophila were harvested at baseline, 18, 48, and 168 h after induction of sepsis and weighed in groups of five flies in triplicate. The weights (0.8 ± 0.053 μg/fly) were unchanged within and between groups over a 1-week period following sepsis. (PDF 67 kb) [file 40635_2016_75_MOESM2_ESM.pdf]
